# Feline enteropathogens and molecular diagnostics: benefits, limitations and clinical applications

**DOI:** 10.1177/1098612X251352746

**Published:** 2025-08-08

**Authors:** Giulia Cattaneo, Katie E McCallum

**Affiliations:** The Queen’s Veterinary School Hospital, Department of Veterinary Medicine, University of Cambridge, UK

**Keywords:** Enteropathogens, intestinal disease, PCR, molecular diagnostics, molecular testing

## Abstract

**Practical relevance:**

Feline enteric disease is a commonly encountered presentation in clinical practice. Interpretation of the clinical relevance of enteropathogens is often misunderstood and can lead to inappropriate case management or overtreatment.

**Clinical challenges:**

The approaches to enteric disease, and the enteropathogens responsible, have proven to be an ever-emerging and challenging area within feline medicine. There are often many difficulties regarding diagnosis, interpretation of results and indications to treat. It is important to understand the aetiopathogenesis of disease, population predispositions and the principles underlying diagnostic testing, including its benefits and limitations, to appropriately manage these cases in clinical practice. Diagnostic testing and treatment should be carried out in a targeted manner only where indicated to do so.

**Evidence base:**

This review provides extensive summaries of the most pertinent feline enteropathogens and diagnostic methods available, as well as their limitations, with a particular focus on molecular testing. The authors have provided their substantiated opinion on how best to approach these cases.

**Global importance:**

An enhanced understanding of feline enteric disease is required not only for improved management of these veterinary patients but also particularly relates to the critical topic of antibiotic stewardship and judicious use of antibiotics, which form the mainstay of treatment for many enteropathogens, but are often used inappropriately in healthy cats testing positive for organisms that are not implicated in enteric disease.

**Audience:**

The target audience for this review encompasses general and specialist practitioners, alongside researchers within this field.

## Introduction

Cats presenting with intestinal disease are often encountered in clinical practice. Many enteropathogens, although associated with diarrhoea, are prevalent (often to a greater degree) in subclinical cats.^1–3^ This is likely to influence diagnostic interpretation and clinical decision-making. The recorded prevalences of disease are also affected by a combination of factors including the diagnostic detection methods used, sample timing and quality. Furthermore, many studies assessing prevalence data fail to provide insight into treatment and outcomes. The detection of a pathogen does not equate to causality and the paucity of robust evidence to suggest that treating these organisms will resolve the clinical signs poses a challenge to interpretation of a positive test.

**Figure:**
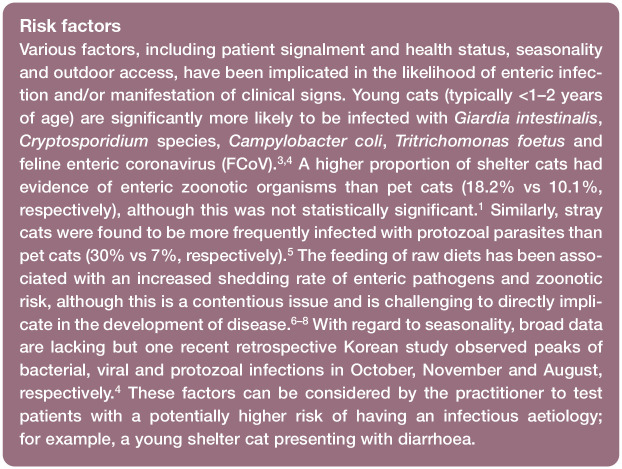


## Feline enteropathogens

**Figure 1:**
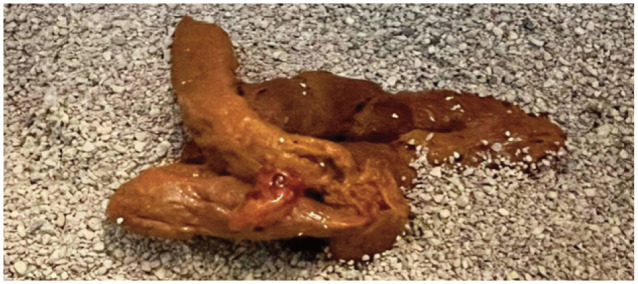
**An example of large intestinal diarrhoea in a cat**. *Courtesy of Arianna Baldini*

**Figure 2:**
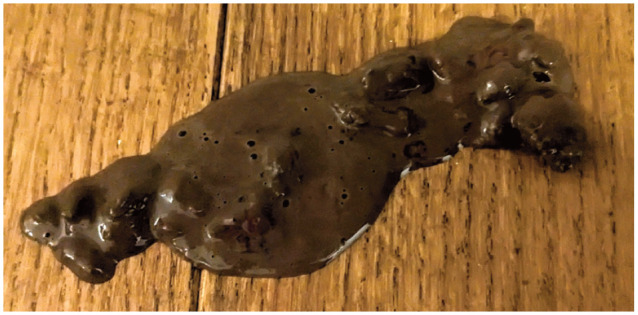
**An example of mixed (large and small intestinal) diarrhoea in a cat**. *Courtesy of Danielle Roussel*

The organisms implicated in feline intestinal disease can be broadly divided into bacterial, viral, protozoal, fungal and parasitic aetiologies. Table 1 provides a summary of each of the organisms implicated, including the most common location of pathology – the large intestine and/or small intestine (Figures 1 and 2 provide examples of large intestinal and mixed diarrhoea observed in the cat), the chronicity of the disease and the diagnostic methods available. The enteropathogens highlighted in Table 1 are discussed individually in more detail and with respect to molecular diagnostic methods in the remainder of this section.

**Table 1:** Summary of organisms implicated in feline intestinal disease including location of pathology, chronicity and detection methods^9–51^

Organism		Small intestinal (SI) vs large intestinal (LI)	Acute vs chronic	Detection methods		
Bacteria	Salmonella species (S enterica^*^ and S bongori)	SI, LI	Acute or chronic	Recommended ✜ Faecal culture ✜ Faecal PCR ✜ Blood culture and/or PCR (if septicaemic)	Complementary technique ✜ FISH (dependent on histopathology)	
	Campylobacter species (C upsaliensis, C helveticus, C coli^*^, C jejuni^*^ and C lari)	SI, LI	Acute or chronic	Recommended ✜ PCR ✜ (± Culture)	Complementary technique ✜ FISH (dependent on histopathology)	Not recommended but available ✜ Faecal microscopy (Gram-stained smear for Camplylobacter-like organisms) – poor sensitivity
	Clostridium species (C difficile^*^, C perfringens^*^ and C piliforme)	SI, LI	Acute or chronic	Recommended Faecal organism detection: ✜ Culture ✜ PCR ✜ ELISA (antigen) Faecal toxin detection: ✜ Cell reverse passive latex agglutination assay ✜ Culture cell cytotoxicity assay ✜ ELISA ✜ PCR (toxin gene)	Complementary technique ✜ FISH (dependent on histopathology)	Not recommended but available ✜ Faecal cytology (endospores)
	*Escherichia coli*	SI, LI	Acute or chronic	Recommended ✜ Faecal PCR ✜ Toxigenic strain bioassays or PCR	Complementary technique ✜ FISH (dependent on histopathology)	Not recommended but available ✜ Routine faecal culture
	Mycobacterium species	SI, LI	Chronic	Recommended ✜ Histopathology (acid-fast bacteria) ✜ Culture ✜ Faecal or tissue PCR ✜ Interferon gamma release assay		
	Yersinia species	SI, LI	Acute or chronic	Recommended ✜ Faecal culture ✜ FISH (dependent on histopathology)	Complementary technique ✜ FISH (dependent on histopathology)	
	*Helicobacter pylori*	Gastric, SI	Acute or chronic	Recommended ✜ Histopathology ✜ Culture ✜ PCR ✜ IHC	Complementary technique ✜ FISH (dependent on histopathology)	
	Anaerobiospirillum species	SI, LI	Acute or chronic	Recommended ✜ Histopathology ✜ PCR		

Organism		Small intestinal (SI) vs large intestinal (LI)	Acute vs chronic	Detection methods	
Viruses	FCoV/FIP virus	SI, LI	Acute or chronic	Recommended FCoV detection: ✜ Faecal/tissue/fluid/blood RT-PCR or qRT-PCR ✜ ICC tissue/fluid (antigen) ✜ IHC tissue (antigen) FIP-specific detection: ✜ Faecal/tissue/fluid/blood S gene RT-PCR for spike protein mutation	Not recommended but available FCoV detection: ✜ Serology (eg, IFAT, ELISA)
	FPV	SI, LI	Acute or chronic	Recommended ✜ Faecal ELISA POC (antigen) ✜ Faecal/blood/vomit/tissue PCR	Not recommended but available ✜ Viral isolation ✜ Electron microscopy (viral particles)
	Astrovirus	SI	Acute	✜ Faecal RT-PCR/qRT-PCR	
	Norovirus	SI	Acute	✜ Faecal RT-PCR/qRT-PCR	
	Rotavirus	SI	Acute	✜ Faecal RT-PCR/qRT-PCR ✜ Electron microscopy (viral particles)	
	Torovirus	SI	Chronic	✜ Faecal RT-PCR/qRT-PCR	
	Bocavirus	SI	Acute	✜ Faecal PCR	
	Picobirnavirus	SI	Acute	✜ Faecal RT-PCR	
	Feline enteric calicivirus	SI	Acute	✜ Faecal RT-PCR ✜ Virus isolation	
	FIV	SI, LI	Chronic	✜ PCR ✜ ELISA POC (antibody)	
	FeLV	SI, LI	Chronic	✜ Serology ✜ PCR (proviral DNA) ✜ ELISA POC (p27 antigen) ✜ IFA (p27 antigen)	
Protozoa	Giardia intestinalis (syn Giardia lamblia; Giardia duodenalis)	SI, LI	Acute or chronic	Recommended ✜ Faecal zinc centrifugal floatation (oocysts) ✜ Faecal ELISA POC (antigen) ✜ Faecal IFA (antigen)	Not recommended but available ✜ Microscopy of motile trophozoites (direct faecal smear) – poor sensitivity ✜ Faecal PCR ✜ Visualisation of organisms in intestinal biopsies
	Tritrichomonas foetus	SI, LI	Acute or chronic	Recommended ✜ Faecal PCR (colonic wash)	Not recommended but available ✜ Colonic biopsy (histopathology) ✜ Direct faecal wet mount (fresh faeces or colonic wash) – poor sensitivity ✜ Faecal culture
	*Toxoplasma gondii*	SI, LI	Acute or chronic	Recommended ✜ PCR/qRT-PCR (eg, faeces, blood, fluid [cerebrospinal fluid, aqueous humour], tissues) ✜ Serology (eg, MAT, ELISA - for systemic infections) ✜ Cytological/histological detection (± IHC)	Not recommended but available ✜ Faecal floatation (oocysts) - poor sensitivity
	*Cryptosporidium species* (Cryptosporidium parvum)	SI, LI	Acute or chronic	Recommended ✜ Faecal IFA (antigen) ✜ Faecal ELISA (antigen)	Not recommended but available ✜ Faecal floatation and oil immersion microscopy with staining (oocysts) – poor sensitivity ✜ Faecal PCR
	Cystoisospora species	SI	Acute or chronic	✜ Direct faecal microscopy (oocysts) ✜ Faecal floatation	
	*Entamoeba histiolytica*	LI	Acute	✜ Direct faecal smears ✜ Electron microscopy (tissue) ✜ IHC	
Fungal	*Pythium insidiosum*	SI, LI	Chronic	✜ PCR (tissue) ✜ Histopathological staining (eg, Gomori’s methenamine silver) ✜ Culture ✜ Serology	
	Histoplasma species	LI	Chronic	✜ Cytology (rectal scrapings or brush samples from colonoscopy) ✜ Histopathological staining (eg, PAS) ✜ Urine EIA (antigen)	

Organism		Small intestinal (SI) vs large intestinal (LI)	Acute vs chronic	Detection methods
HELMINTHS (nematodes)	Strongyloides species (roundworm)	SI, LI	Acute or chronic	✜ Direct faecal microscopy (larvae) ✜ Sedimentation ✜ Faecal Baermann technique ✜ Tissue and faecal PCR
	Ancylostoma species (hookworm)	SI	Acute or chronic	✜ Faecal floatation (ova) ✜ Faecal PCR ✜ ELISA (coproantigen)
	Toxocara cati (roundworm)	SI	Acute or chronic	✜ Faecal floatation (ova) ✜ Faecal PCR ✜ ELISA (coproantigen)
	Toxascaris leonina (roundworm)	SI	Acute or chronic	✜ Faecal floatation (ova)
	Uncinaria stenocephala (hookworm)	SI	Acute or chronic	✜ Faecal floatation (ova)
	Trichuris species (whipworm)	LI	Acute or chronic	✜ Faecal floatation (ova) ✜ Faecal PCR
HELMINTHS (cestodes)	*Dipylidium caninum*	SI	Acute or chronic	✜ Direct faecal microscopy ✜ Faecal floatation (ova)
	Spirometra species	SI	Acute or chronic	✜ Direct faecal microscopy (ova)
	*Diphyllobothrium latum*	SI	Acute or chronic	✜ Direct faecal microscopy (ova)
	Taenia species	SI	Acute or chronic	✜ Direct faecal microscopy (ova) ✜ Faecal floatation ✜ Faecal PCR
HELMINTHS (trematodes)	*Nanophyetus salmincola*	SI	Acute or chronic	✜ Direct faecal microscopy (ova)
	*Platynosomum fastosum*	SI	Acute or chronic	✜ Direct faecal microscopy (ova) ✜ Faecal floatation (ova)

The organisms highlighted in pink are discussed in more detail within the text and with respect to molecular diagnostic methods. Of these, the species with asterisks (*) are thought to be the enteropathogens more commonly implicated in feline enteric disease. Tests recommended for diagnosis of the organisms discussed are specifically indicated, either alone or in combination (please refer to the text) EIA = enzyme immunoassay; FCoV = feline enteric coronavirus; FeLV = feline leukemia virus; FIP = feline infectious peritonitis; FISH = fluorescence in situ hybridisation; FIV = feline immunodeficiency virus; FPV = feline panleukopenia virus; ICC = immunocytochemistry; IFA = immunofluorescence assay; IFAT = immunofluorescent antibody test; IHC = immunohistochemistry; MAT = microscopic agglutination test; PAS = Periodic acid–Schiff; POC = point-of-care; qRT-PCR = quantitative reverse transcription polymerase chain reaction

The organisms highlighted in pink are discussed in more detail within the text and with respect to molecular diagnostic methods. Of these, the species with asterisks (*) are thought to be the enteropathogens more commonly implicated in feline enteric disease. Tests recommended for diagnosis of the organisms discussed are specifically indicated, either alone or in combination (please refer to the text) EIA = enzyme immunoassay; FCoV = feline enteric coronavirus; FeLV = feline leukemia virus; FIP = feline infectious peritonitis; FISH = fluorescence in situ hybridisation; FIV = feline immunodeficiency virus; FPV = feline panleukopenia virus; ICC = immunocytochemistry; IFA = immunofluorescence assay; IFAT = immunofluorescent antibody test; IHC = immunohistochemistry; MAT = microscopic agglutination test; PAS = Periodic acid–Schiff; POC = point-of-care; qRT-PCR = quantitative reverse transcription polymerase chain reaction

### Salmonella species

The two known Salmonella species are Salmonella enterica (further divided into six subspecies, including S enterica subspecies enterica, which is implicated in feline intestinal disease) and Salmonella bongori; these are Gram-negative, motile, facultative anaerobic bacilli. Clinically affected cats present with non-specific signs including pyrexia, malaise, abdominal pain, diarrhoea and vomiting.^11^ ‘Songbird fever’ has been described in cats in Mediterranean countries following ingestion of migrating songbirds carrying S enterica serotype Typhimurium (S Typhimurium) and is characterised by acute, seasonal, febrile diarrhoea.^9^ In severe cases of infection with Salmonella species, sepsis can manifest by invasion and translocation from the gut lumen via transmembrane proteins expressed by enterocytes.^9,52^ Salmonella species are ubiquitous organisms and have been isolated in both diarrhoeic (0–8.6%) and non-diarrhoeic (0–4%) cats.^1,3,11,53–56^ Data derived predominantly from dogs postulate that the feeding of raw diets, or a recent history of probiotic or antibiotic use, could be implicated in the isolation of Salmonella species.^8,52,54,57^ However, some of these studies involve low numbers of animals, particularly with regards to data pertaining to probiotics and antibiotics; therefore, difficulties in the establishment of causality must be carefully considered before drawing such conclusions in cats.

There is limited information regarding the most commonly identified Salmonella species in cats; although Salmonella species were only detected in 2/199 cats in one study, serotyping was consistent with identification of S enterica in both these cases.^1^ S enterica was isolated in 0.8% of cats by PCR in another study;^55^ however, it should be noted that other species were not included in the testing panel, which precludes the usefulness of these data.

Commercial PCR tests for Salmonella species identification are now available and reported in the literature.^55^ Regarding the diagnostic method of choice, a combination of faecal PCR and culture (as well as blood PCR and/or culture in a septicaemic animal) should be employed. Previous recommendations^11^ of faecal enrichment culture followed by PCR are still considered the gold standard. Positive PCR samples can undergo further selective enrichment culture for organism identification,^11^ with the additional benefit that antibiotic sensitivity testing may also be performed on culture samples.

### Campylobacter species

Campylobacter species detected in cats and dogs, such as Campylobacter jejuni, Campylo -bacter upsaliensis, Campylobacter helveticus, Campylo bacter lari and C coli, are Gramnegative, microaerophilic, curved motile rods;^2,9,11,58,59^ C helveticus and C upsaliensis are the most common species identified in cats irrespective of the presence or absence of clinical signs.^11,60^ In a species-specific PCR assay performed on the faeces of 47 commercially reared healthy cats, 83% of Campylobacter species-positive isolates were identified as C helveticus, 47% as C upsaliensis and 6% as C jejuni;^2^ however, not all these species are implicated in disease. C coli has been associated with diarrhoea in kittens^9^ and detected via fluorescence in situ hybridisation (FISH) within the duodenal mucosa of cats with neutrophilic inflammatory bowel disease (IBD).^14^ In another study, a higher prevalence of C jejuni was found in diarrhoeic cats compared with other Campylo bacter species.^58^

The prevalence of Campylobacter species in cats is variable and risk factors for shedding include intensive housing or shelter environments, young age, access to the outdoors and seasonality.^11,56,60,61^ In several studies, overall prevalence based on PCR ranged from 42.9% to 56.5%.^3,56,62^ Campylobacter species have been isolated from 9.6–47.6% of diarrhoeic vs 18–27.8% of non-diarrhoeic cats;^3,56,59^ the similar isolation rates likely reflect the fact that Campylobacter species isolated from cats are predominantly non-pathogenic.^3^ Despite this, a higher prevalence of Campylobacter species (52%) in shelter cats with diarrhoea has been reported.^56^ The prevalence data are likely to be impacted by whether campylobacteria were diagnosed by culture or PCR as the latter has been demonstrated to show higher sensitivity in the detection of Campylobacter species (13.2% vs 56.5%, respectively).^3,58^

While commercial PCR techniques allow for speciation^62^ (important specifically for C coli and C jejuni), available routine culture tests do not tend to provide this information. Based on the current evidence, the authors believe that C coli and C jejuni have a potential pathogenic role in feline diarrhoea and would only recommend testing for these organisms through the use of PCR techniques (or a combination of culture and PCR).

### Clostridium species

Clostridium species with pathogenic potential include Clostridium (Clostridioides) difficile and Clostridium perfringens; however, these species are common in cats, and data to support their pathogenicity are limited. A number of non-pathogenic species also exist. Clostridium species are Gram-positive anaerobic, toxinproducing bacilli that can be observed in the vegetative (‘actively growing’) and spore (‘dormant’) forms. Spores are highly resistant in the environment and important as a means of transmission,^11^ converting to the vegetative forms when intestinal conditions are favourable. Certain strains of C difficile produce toxin A (TcdA, enterotoxin) and toxin B (TcdB, cytotoxin),^63^ although binary toxin (CDT) has also been isolated (albeit with unclear significance). TcdA and TcdB are commonly produced together and are thought to be implicated in intestinal disease.^10,11^ C perfringens can be divided into five biotypes (A–E) depending on the possession of major toxin genes (alpha, beta, epsilon and iota).^11,64^ The majority of enterotoxigenic strains isolated in cats belong to Type A and produce the enterotoxin CPE (gene cpe), although production of beta-2 toxin may also have a role to play in virulence. Alpha toxin (gene cpa) is present in all strains and therefore its role in virulence is also questioned.^11^ Risk factors for colonisation with C difficile are similar to those in humans, including administration of antimicrobials or immunosuppressive agents.^63,65,66^

The prevalence of C perfringens in healthy cats appears to be lower than dogs.^11^ Overall prevalence rates based on culture and/or toxin testing were reported to be 19–63%,^1,11,67,68^ with prevalences of 14–86% in diarrhoeic cats^3,53,56,67,69^ compared with 38–86% in non-diarrhoeic cats.^1,3,53,67^ Of 80 shelter cats with diarrhoea, the most common enteropathogen detected by faecal PCR was C perfringens (81%), although the ubiquitous alpha toxin gene was tested.^56^ The overall prevalence of C difficile in cats (clinical or subclinical) was reported to be 6–9.4%,^63,68^ with 50% showing clinical signs of diarrhoea in one study.^68^ Interestingly, in humans and dogs, C difficile has been linked to chronic enteropathies through mechanisms other than the aforementioned direct enteropathogenic effects; the presence of C difficile was associated with increased dysbiosis index and bile acid dysmetabolism due to a reduction in Peptacetobacter (Clostridium) hiranonis abundance.^70^ There is currently little evidence to support this association in dogs, and it has not yet been proven in cats.

The diagnosis of Clostridium species, in particular C difficile, requires a combination of organism and toxin detection, with cell cytotoxicity assays considered the gold standard for C difficile infections; however, these are not routinely used. Combining faecal culture and/or antigen testing with ELISA toxin detection is recommended to diagnose C difficile,^71^ whereas combining CPE toxin detection by ELISA and PCR is recommended to diagnose C perfringens.^11,72^

The role of clostridial toxins in intestinal disease, however, is disputed; CPE was detected in both diarrhoeic (4.1%) and non-diarrhoeic cats (1.9%),^3^ while C perfringens alpha toxin was detected in 42–56.6% and 50% of cats with and without diarrhoea, respectively.^53,55^ C perfringens alpha toxin was associated with increased risk of diarrhoea in cats coinfected with FCoV.^46^ In another study,^73^ the diagnostic value of a faecal panel in diarrhoeic dogs was deemed to be low, although some association of acute haemorrhagic diarrhoea with the presence of CPE and TcdA (identified by ELISA) was found. More recent studies suggest an association between C perfringens strains encoding poreforming toxin genes netE and netF and the development of acute haemorrhagic diarrhoea syndrome (AHDS) in dogs,^74,75^ though this has not yet been reported in cats. Despite this, there were no differences in recovery time or outcome between netF-positive and netF-negative dogs with AHDS and therefore there is no indication for targeted treatment strategies currently.^74^ Of cats found to be positive for C difficile on faecal PCR, only 14.3–34.8% of these were toxigenic strains.^63,69^ This raises the question of whether detection of toxins can be reliably correlated with disease and, ultimately, their diagnostic value.

In summary, when detecting Clostridium species, the authors recommend avoidance of interpretation of organism detection alone and, where possible, molecular diagnostics for toxin detection should be employed on a case-by-case basis. Toxins that are known to be ubiquitous, such as C perfringens alpha toxin (cpa), must be interpreted with caution.

### Escherichia coli

E coli is a Gram-negative bacillus observed as part of the commensal intestinal flora; however, enteropathogenic (EPEC), enterohaemorrhagic (EHEC) and enterotoxigenic (ETEC) strains exist, which may manifest as acute or chronic diarrhoea.^9,76^ Shiga-toxin producing E coli (STEC) have been documented in cats via PCR detection and provide a potential reservoir of infection for haemolytic uraemic syndrome in humans.^77^ E coli is thought to play a role in the pathogenesis of feline chronic enteropathies: a higher abundance of mucosal bacteria including Enterobacteriaceae, E coli and Clostridium species (as determined by FISH) were strongly associated with the presence and severity of intestinal inflammation in cats.^78^ In cats with chronic enteropathies, intestinal dysbiosis was characterised by increases in E coli populations (detected by real-time [quantitative] PCR [qPCR]), among other alterations in faecal microbiota,^79^ although cause and effect remains to be determined and is likely to be multifactorial.

In one study, faecal bacterial cultures (including E coli) failed to distinguish between healthy dogs vs those with chronic diarrhoea and there was no agreement with the dysbiosis index.^80^ Faecal culture is therefore not recommended for diagnosis of disease mediated by E coli, and PCR is instead recommended to identify the gene(s) associated with pathogenicity.^10^

### Feline enteric coronavirus and feline infectious peritonitis virus

FCoV is an enveloped RNA virus with spike proteins that provide a means of entry into cells. This virus is transmitted via the faeco-oral route, has a particular tropism for enterocytes and typically manifests as transient and mild diarrhoea. Seroprevalence is higher in multicat households. Spontaneous mutation, for example of the spike (S) protein gene, confers a change of tropism to macrophages/monocytes, which propagates infection to extraintestinal tissues and can ultimately give rise to feline infectious peritonitis (FIP). This predominantly results in the development of pyogranulomatous lesions with or without vasculitis, manifesting as a wide range of clinical signs; although it is broadly referred to as a ‘wet’ (effusive) or ‘dry’ (non-effusive) form, there can be some overlap between these two categories.^81,82^

In a retrospective study assessing the faecal samples of 1620 diarrhoeic cats, FCoV was the most common pathogen isolated by PCR (29.37%).^4^ Furthermore, FCoV has been detected in 40–87% of diarrhoeic^46,53,55,56^ vs 36–59% of non-diarrhoeic cats.^46,53^ The outcomes of primary FCoV infection include quick cessation or absence of shedding (due to innate resistance, ~5%), low-level intermittent shedding for a few months (~70–80%) or persistent and long-term high-level shedding (~10–15%). It is estimated that approximately 5–12% of cats infected with FCoV develop FIP.^19,81–83^

Despite the association of FCoV with gastrointestinal signs, the high prevalence of this pathogen in the feline population poses a challenge to establishment of causality and raises the question as to whether it may be a predominantly coincidental finding. Frequent coinfections with other enteropathogens may further complicate this picture; while FCoV infection was significantly associated with diarrhoea (odds ratio 5.01) in one study, 95% of FCoV-positive diarrhoeic cats had coinfections with other enteropathogens.^46^ Despite this, FCoV-positive cats with coinfections in this same study were actually deemed no more likely to have diarrhoea than FCoV-positive cats without coinfections (P = 0.455), which may support causality of FCoV as a true enteropathogen.^46^ Similarly, it was found that diarrhoeic cats were equally infected with one or more enteropathogens (84%) when compared with cats with normal faeces (84%) and only feline coronavirus was significantly more prevalent in diarrhoeic cats (58%) than cats with normal faeces (36%).^53^ Alongside the uncertain role of coinfections and causation of diarrhoea, the reader should consider that other complicating factors, such as FCoV intermittent shedding and reinfection (particularly in multicat households), are likely to influence the results of testing methods and their interpretation on an individual basis. Of the aforementioned studies,^46,53,55,56^ there were no data provided on treatment and outcome of the cases; self-limiting clinical signs may have spontaneously resolved or responded to non-specific treatment.

Despite the high prevalence and self-limiting nature of clinical signs, the authors perform FCoV testing (serology and faecal PCR) as it can provide clinically relevant information in selected cases such as multicat households, shelters and catteries where identification of chronic shedders is key to minimising viral load in the environment.^84^ Some correlation between degree of shedding and antibody titres has been observed, although cats may remain seropositive for prolonged periods of time beyond the cessation of shedding. Obtaining at least five consecutive monthly negative faecal PCR tests, or proving seronegativity, have previously been suggested to confirm the cessation of shedding;^83^ in light of the known persistence of seropositivity, chronic shedders should ideally be identified on the basis of serial faecal RT-PCR tests.^85^ The reader is also referred to the ‘AAFP/EveryCat feline infectious peritonitis diagnosis guidelines’^19^ for further information on diagnostic testing and interpretation. Although the use of combination diagnostics (including PCR and serology) is commonly employed clinically, immunohistochemistry and FCoV antigen detection is still considered the gold standard for the diagnosis of FIP.^19,86^

### Feline panleukopenia virus

Feline panleukopenia virus (FPV) is a non-enveloped single-stranded DNA virus spread through the faeco-oral route. Clinically affected animals typically display panleukopenia, thrombocytopenia and anaemia due to lymphoid and bone marrow involvement and severe, often haemorrhagic, enteritis due to destruction of intestinal crypt cells. Signs may also manifest as cerebellar hypoplasia (in utero or neonatal infection) and abortion.^87^ Death is usually as a result of complications such as septicaemia, dehydration and disseminated intravascular coagulation.^88^ Vaccination is considered a preventive measure, with antibody titres correlating with protection.^88,89^

Point-of-care (POC) canine ELISAs detecting antigen (canine panleukopenia virus [CPV]-2a strains and/or FPV antigen) in faeces, alongside faecal/blood/vomit PCR testing (the latter two being useful if there is no diarrhoea present), are important methods of diagnosis in cats and are preferred over serological tests due to their inability to distinguish between vaccination and infection, although convalescent titre testing is possible.^45,87,88,90^ In one study, the sensitivities and specificities of various POC ELISA tests in cats ranged from 50% to 80% and 94% to 100%, respectively.^90^ A recent study^91^ compared the results of a POC ELISA test (faeces or rectal/anal swabs) and PCR (anal/rectal swabs and vomit) using faecal PCR as the reference standard; the sensitivities of faecal and swab POC ELISA were inferior (55% and 30%, respectively) to swab and vomit PCR (77% and 100%, respectively). Specificity was 96–100% for all sample and test types; however, some care must be taken when interpreting POC ELISA and/or PCR results due to the risks of false positives (vaccine virus shedding reported 2–4 weeks post-vaccination)^92,93^ and false negatives (delay between viraemia and faecal shedding, intermittent shedding or cessation of viral shedding by the time clinical signs appear/testing is performed, faecal virus binding antibodies and making them unavailable for the assay).^45,90,94,95^

Cats in Asia have been shown to be commonly infected (>80% of isolates) with the canine counterpart (CPV), the genetic sequence of which is very similar to FPV and differs by only 2%.^88,95,96^ CPV was demonstrated in >30% of faecal samples of healthy shelter cats via PCR in the UK;^97^ however, these strains have been generally less frequently isolated in other parts of Europe and the USA.^88,98^ CPV-2a, 2b and 2c strains are all able to infect cats but clinical illness, although indistinguishable, is rare.^87,95^ Shedding of FPV can be prolonged and cats are considered to be important reservoirs of infection for both cats and dogs.^88,97^

### Giardia intestinalis

G intestinalis (syn lamblia/duodenalis) is a flagellated protozoan parasite transmitted by the faeco-oral route; ingested oocysts excyst to become trophozoites, which multiply within the small intestinal mucosa leading to faecal excretion of oocysts.^9,51^ Seven genotypes have been identified (A–G), with Assemblage F primarily infecting cats.^9,99^ Giardia species have been detected in 5–20.6% and 1–8% of cats with and without diarrhoea, respectively,^1,53,55,56^ including 0–9.2% of healthy cats in shelters^1,53,100^ compared with 8.3–20% of shelter cats with diarrhoea.^1,53^ These results may suggest a trend towards higher prevalence in diarrhoeic cats and its significance as an enteropathogen; however, it is not possible to effectively compare prevalences from different studies to make this determination. In a recent meta-analysis, the prevalence rate for G intestinalis in cats was 12%, with a greater prevalence in clinical vs subclinical animals and younger animals (<6 months) and a lower risk in client-owned pets.^101^ High housing density^102,103^ and seasonality (dogs)^104^ have also been found to affect prevalence of disease.

The diagnostic method used between studies appears to have an impact on reported prevalence; ELISA, immunofluorescence assay (IFA) and PCR techniques have reported greater sensitivities when compared with microscopy.^101,105,106^ However, one feline study documented faecal floatation to be a useful method of detection with comparable sensitivity and specificity to the immunoassay (POC Giardia species), albeit with the latter more practical to use.^107^ Combination testing of faecal floatation and faecal antigen immunoassay has been recommended for diagnosis in cats with diarrhoea (97.8% sensitivity), with PCR not recommended as a sole means of diagnosis due to false-negative results secondary to PCR inhibitors.^107,108^

### Tritrichomonas foetus

T foetus is a flagellate protozoan that only exists in the trophozoite form. Transmission is via the faeco-oral route and results in colonisation of the colonic mucosa with predominantly large intestinal diarrhoea and anal irritation.^10,26^ Although the disease is generally self-limiting, some cats develop chronic or intermittent clinical signs that can spontaneously resolve within 2 years (median 9 months; range 5 months to 2 years). The manifestation of clinical signs is thought to be dependent on host immune response, endogenous microflora, pathogenicity of the strains and coinfection (eg, G intestinalis).^10^ Many cats develop persistent infections, with recrudescence during periods of stress leading to sub-clinical shedding, which demonstrates these cats as a reservoir of infection.^26,109^

Infected cats are predominantly young (typically <1 year of age) with a recent history of diarrhoea and from multicat households, shelters, catteries or breeding colonies. There is also a predisposition for pedigree breeds such as the Siamese or Bengal.^25,102,110^ The prevalence of T foetus in cats with diarrhoea ranges between 2% and 18.8%^3,55,56,110,111^ compared with 0% in non-diarrhoeic cats.^3^ Interestingly, the prevalence of T foetus specifically in cattery and/or shelter cats has been found to be up to 31% (with a low prevalence reported in Australia) when compared with 0% in pet cats.^53,102,111,112^

Detection by PCR has a superior sensitivity than faecal culture (55%) or direct faecal wet mount (⩽14%) and is the authors’ diagnostic test of choice.^26^ T foetus PCR is more likely to be positive in diarrhoeic stools, and a colonic flush technique is recommended to improve PCR sensitivity.^10^ For T foetus, the European Advisory Board on Cat Diseases only recommends the treatment of cats that demonstrate both diarrhoea and positive testing.^25,26^

### Toxoplasma gondii

T gondii is a protozoan parasite for which cats are the definitive host and other mammals the intermediate hosts; it is spread via the faeco-oral route (faecal shedding of oocysts) or ingestion of tissue cysts (eg, ingestion of the intermediate host) and typically results in subclinical infection or transient, self-limiting diarrhoea in newly exposed cats with enteroepithelial replication. Tachyzoites or bradyzoites are formed during the extraintestinal infection life cycle; tachyzoites predominate in an active infection and replication can be controlled with an adequate immune response, whereas bradyzoites (tissue cysts) are a slowly dividing form that can reactivate under favourable conditions such as immunosuppression.^15,17,27,113,114^ Clinical toxoplasmosis develops following dissemination and intracellular replication of tachyzoites, with central nervous system, muscular, ocular and pulmonary involvement most common.^27^ The prevalence of T gondii faecal shedding (PCR) in cats with diarrhoea was found to be 1%.^55^

Faecal PCR has been reported to be more sensitive than microscopy to detect shedding, with as many as 20% of cats without clinical signs being DNA positive.^28,115,116^ Similarly, T gondii has been detected by multicopy target PCR with increased sensitivity when compared with conventional microscopy (8.5% vs 3.5%, respectively) on faecal samples in cats.^117^ Differentiation of coccidian oocysts under microscopy can also be challenging if they share similar morphological characteristics.^118,119^ The authors believe that faecal testing in cats is of low yield given the largely subclinical nature of the intestinal phase of infection. On the other hand, although definitive diagnosis of disseminated toxoplasmosis requires cytological or histopathological demonstration of T gondii organisms, the sensitivity is generally low and therefore this can be combined with PCR assays (of blood or affected tissue) and convalescent serology to achieve a diagnosis in individuals with compatible clinical signs.^15–17 ,120,121^

### Cryptosporidium species

Cryptosporidium parvum is an obligate intracellular protozoan parasite transmitted through the faeco-oral route. It is more common in cats than dogs and is generally subclinically shed, although it has been associated with self-limiting diarrhoea. Stress, coinfection or immunosuppression may lead to clinical manifestation of signs including severe haemorrhagic diarrhoea;^9^ in one study, the reappearance of Cryptosporidium species oocysts was observed in the faeces of 2/4 cats following prednisolone treatment in cats experimentally infected with T foetus.^122^ Cryptosporidium species were detected in 10–24.4% and 4.6–20% of cats with and without diarrhoea, respectively,^53,55,123^ while in other studies the total prevalence (irrespective of clinical signs) in cats was found to be 4.6–12.1%.^1,100,123,124^

Immunoassay techniques (faecal IFA and ELISA) are highly sensitive, specific and thought to be the diagnostic method of choice for Cryptosporidium species in cats.^1,18,125^ Cytological examination and faecal floatation are not recommended due to poor sensitivity.^125^

**Figure:**
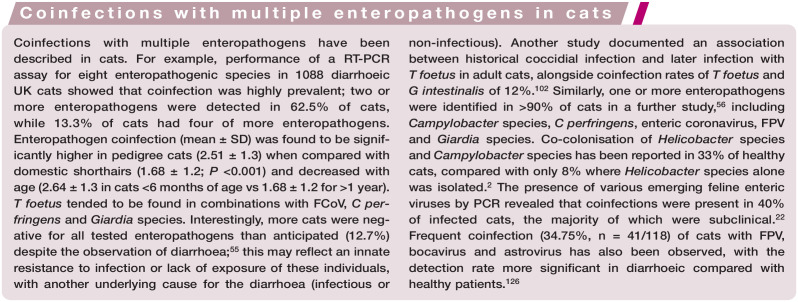


## Molecular testing in feline intestinal disease

### What are the indications for molecular testing in feline intestinal disease?

Molecular testing methods in feline intestinal disease, although useful and with significant potential, are not as simple to interpret as it may initially seem; requesting these tests in our patients only where it is indicated to do so is therefore important. A summary, including in relation to molecular testing, which is suggested as a standard for specific pathogens, can be found in the box ‘Indications for feline enteropathogen testing’.

For individuals that are subclinical or display very mild, self-limiting disease, the use of molecular testing is arguably futile and will be unlikely to change the treatment plan, which should be primarily supportive, if required at all. Testing for enteropathogens in subclinical individuals not only proves to be an unnecessary cost to the client but may also yield misleading or confounding results with resultant inappropriate treatment.

Given that many bacterial or protozoal diseases are treated with antibiotics and antibiotic resistance of enteropathogens is an emerging and significant public health concern,^129–131^ we are obligated to judiciously use these drugs so as to reduce the drive for resistance and therefore maximise chances of treatment success. Symptomatic treatment strategies for acute diarrhoea in veterinary patients only support the use of preventive antibiosis in cases displaying severe, intractable disease with evidence of sepsis or at high risk of bacterial translocation that are poorly or non-responsive to supportive management (ie, intravenous fluid therapy, antiemetics, etc).^71,127^ Similarly, regarding FCoV, concerns over the development of resistance to antiviral agents, the generally self-limiting or subclinical nature of clinical signs and low potential for transformation to FIP would discourage prophylactic treatment despite recent data to support this practice.^132^

Another important, emerging topic (beyond the scope of this review) is the association of the use of antibiotics with the development of dysbiosis of the intestinal microbiota, both in humans and animals.^133–135^ Although there is still much uncertainty regarding the consequences this may have, there is already evidence to support the development of IBD secondarily to dysbiosis.^136–139^ It is also difficult to know what effect or interactions, if any, dysbiosis may have on the virulence of resident microflora. As previously mentioned, certain enteropathogens have already been associated with dysbiosis;^70,78,79^ however, the relationship between these remains unclear.

For many cats with chronic enteropathies whereby other extraintestinal or intestinal triggers, including infectious agents, have been excluded or are deemed an unlikely cause, dietary modification is an important consideration. Chronic enteropathies in cats are broadly categorised into: food-responsive enteropathies, idiopathic IBD (steroid-responsive enteropathies) and alimentary small-cell lymphoma.^140^ Exclusive hydrolysed or novel protein dietary trials form the mainstay of treatment for food-responsive enteropathies and/or IBD and often require multiple trials before successful outcomes are achieved.^140,141^ The use of probiotics in this disease process has not been substantiated in cats,^140^ although they may still be considered. The reader is referred to recent publications for further information regarding diagnosis and treatment of feline chronic enteropathies.^140,142^

**Figure:**
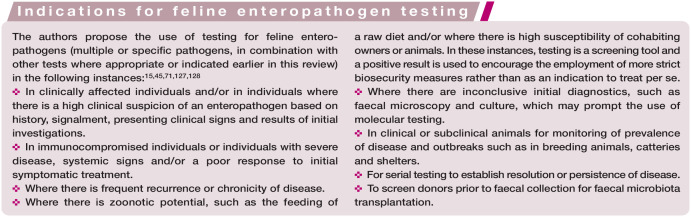


**Figure:**
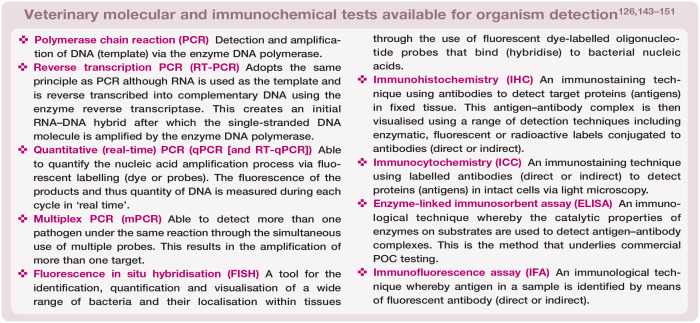


### Molecular testing methods in feline intestinal disease

A definition and outline of the key molecular, as well as immunochemical, tests available for use in the diagnosis of feline intestinal disease can be found in the ‘Veterinary molecular and immunochemical tests available for organism detection’ box. Having an understanding of the processes and pitfalls of the diagnostic tests available is crucial in being able to interpret their results. As a commercially available, sensitive and specific method for screening several enteropathogens in cats,^55^ PCR will be the main focus of the discussion of molecular testing methods in this review (this technique is also described widely in the diagnosis of human infectious gastroenteritis).^152^ The further molecular testing method of FISH is also discussed in the box that appears later on.

**Figure 3:**
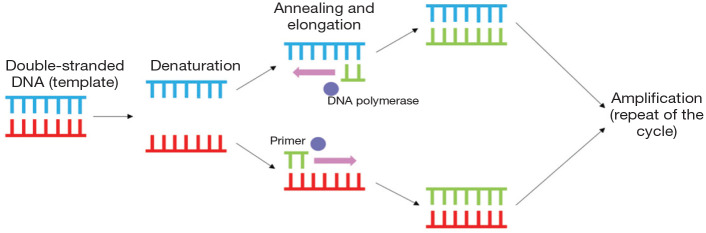
The principle of polymerase chain reaction (PCR), including the denaturation, annealing, elongation and amplification stages

As well as being both sensitive and specific for a range of pathogens, additional benefits to PCR (the process for which is shown in Figure 3) include the need for only small volumes of sample, its utility on samples that cannot be cultured (eg, formalin-fixed tissues) and its ability to detect species that are not culturable.^144,153^ The sensitivity and specificity may be further improved through the targeting of multicopy genes; for example, detection of T gondii oocysts in cat faeces.^117^ It is also possible to detect and distinguish between more than one pathogen concurrently via multiplex PCR (mPCR); FPV, feline bocavirus and feline astrovirus were simultaneously detected with a coincidence rate of 100% when compared with routine PCR in one study.^126^

### Challenges of interpreting results of molecular diagnostic testing in feline intestinal disease

The challenges of interpreting the results of molecular testing are summarised in the ‘Challenges of interpreting molecular testing results’ box and are expanded on in the text below.

**Figure:**
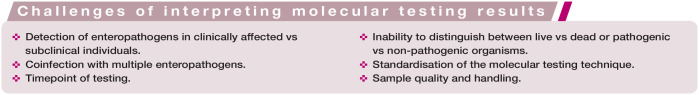


#### Clinically affected vs subclinical individuals

The previously discussed detection of enteropathogens or their toxins in both clinically affected and subclinical cats complicates interpretation of molecular diagnostic testing as it can be challenging to establish a causal relationship with disease. For example, the high prevalence of enteropathogens (eg, E coli, C perfringens and Campylobacter species) in healthy animals is a clear limitation of faecal PCR.^3,9,11,25^

#### Coinfection with multiple enteropathogens

Coinfection with enteropathogens provides further difficulties; positivity for multiple pathogens does not necessarily establish causality and therefore it may be more accurate to term these as ‘co-carriage’. This difference may reflect colonisation rather than association with disease.^56^ However, it is likely that the presence of one pathogen may, in some cases, lead to the potentiation of disease by another (ie, ‘symbiotic’ relationship) through mechanisms such as alteration to the microbiome, reduction in local host immune responses and intestinal epithelial disruption.^55,122^ In one study whereby four cats were experimentally inoculated with T foetus, coinfection with Cryptosporidium species was found to result in exacerbation of transient diarrhoea.^122^ Similarly, persistent C parvum infection was thought to be associated with failure of fenbendazole treatment to eliminate Giardia species infection in cats.^154^ Therefore, it may be important to test for multiple, potentially synergistic pathogens so as to maximise chances of treatment success. However, one recent study found no statistically significant difference in median faecal score or median faecal FCoV load between FCoV-positive cats with and without coinfection with potential enteropathogens.^46^

#### Timepoint of testing

The timing of testing is important in the interpretation of results. For example, intermittent shedding of enteropathogens is another significant limitation of molecular (and other) testing and can yield false-negative results.^5,144^ In fact, serial stool sampling was found to increase the sensitivity of faecal floatation for G intestinalis diagnosis in humans, dogs and cats.^155^ Therefore, the performance of serial (or pooled) stool sampling may aid in improving sensitivity of faecal enteropathogen testing. Recent vaccination can be a risk factor for false-positive results; for example, vaccination with modified live parvovirus has been associated with false-positive faecal antigen ELISA and PCR tests.^92,93^

#### Distinguishing between live vs dead and pathogenic vs non-pathogenic organisms

A major limitation of PCR techniques is that they are generally unable to distinguish between live and dead organisms.^28,144^ However, qPCR plays a role in interpreting the significance of the presence of a pathogen, with a high quantification potentially indicating a clinically significant or active infection. Attempts to circumvent this inability to discriminate between live and dead organisms by targeting viable cells with PCR have been previously described;^156,157^ however, this is not the current standard protocol. Viral cell cultures may be useful in PCR-positive samples to determine whether viable, infectious virus is present, as has been demonstrated with par-vovirus.^97^ Detection of a pathogen on PCR therefore needs to be interpreted with caution and in the light of other clinical variables or faecal testing results.

A further complicating factor for interpretation is that the amplification of a particular gene by PCR does not necessarily correlate with expression and/or virulence, as these are dependent on multiple factors relating to the organism and host.^9,11^ Virulence of Salmonella species, for example, cannot be determined by the presence or absence of virulence genes as these are ubiquitous.^9,11^ As another example, while the correlation of C difficile toxin gene detection by PCR and toxin detection by enzyme immunoassay in humans was found to be 90.6%, with direct detection of toxin-encoding genes by PCR therefore deemed a reliable tool for the detection of toxigenic strains,^158^ as discussed earlier, the presence of clostridial species toxins is not always associated with disease.^3,53,73^ Positivity for pathogenicity markers or toxin bioassays when detecting E coli also does not prove causation.^9,11^ There are some cases, however, where results may suggest expression and/or virulence. For instance, detection of clostridial species toxins by cell cytotoxicity assays or ELISAs may aid in the interpretation of PCR detection of organisms,^11,72^ with the combination of C perfringens enterotoxin detection by ELISA with PCR detection of enterotoxigenic strains being recommended to facilitate the diagnosis of C perfringens-associated diarrhoea.^11^ The use of qPCR alone may also give an indication of the clinical relevance of an organism, with increased numbers of a virulence trait gene potentially supportive of higher organism virulence and therefore significance in the disease process.^159^

A further example of the difficulties of distinguishing between pathogenic vs nonpathogenic organisms relates to FIP. The majority of the available detection methods for FCoV (including serology, FCoV RT-PCR/RT-qPCR, immunocytochemistry and immunohistochemistry) are unable to distinguish between FCoV and FIP virus and are therefore non-specific, although identification of virus in extraintestinal tissues and/or high viral loads may increase the index of suspicion for FIP. More recently, qRT-PCR (on blood, fluid, tissue or faeces) targeting the S gene mutation was developed; although this is more specific to the FIP virus, the sensitivity is lower than conventional PCR because not all cats with FIP will carry this mutation.^19,160,161^

#### Standardisation of the molecular testing technique

PCR success is highly dependent on the technique and, importantly, its standardisation; this is likely to vary greatly between laboratories, however, and is expected to provide diagnostic challenges and also affect prevalence data.^152^ When investigating factors influencing the success rates of PCR, several reaction and technical factors, including denaturing, detection reagents, target gene selection, quantitative calibration, extraction methods and annealing, contribute to yield and PCR result variability.^147,162^ The functioning of the PCR technique also rests upon careful probe design; the detection of a pathogen requires a specific probe and there is no one standardised sequence or target for each.^163^ The variation in results due to this is supported by previous investigations whereby sequence variations in primer binding sites were frequently responsible for failure to detect E coli STEC strains^164^ or TcdA gene.^165^ Genetic plasticity of organisms such as E coli may be another concern, influencing the interpretation of pathogenicity.^163^ There can also be competition between primers and reaction conditions in mPCR, therefore affecting sensitivity and specificity^126^ alongside non-specific sequence amplifications.^166,167^

The above considerations highlight the importance of the utilisation of laboratories that employ strict standardisation and quality control measures for their tests. Any serial testing should be submitted to the same laboratory where possible to limit variability and enable more meaningful comparisons between test results.

#### Sample quality and handling

The quality and quantity of pathogen in the sample will depend on the sample itself, collection technique, storage and processing, and can result in false positives (due to contamination) and false negatives if suboptimal. For example, many compounds found in faeces such as bile salts, bilirubin and complex polysaccharides, and even cat litter, have been shown to interfere with PCR analysis, and therefore the genetic material must be adequately purified. Prior antibiosis and an inappropriate sampling site can affect PCR yield as well.^10,17,144,152,168–170^ Optimal sample collection is important; for example, T foetus PCR is more likely to be positive in diarrhoeic stools and a colonic flush is the recommended technique to improve PCR sensitivity.^10^ Regarding storage, faecal samples were found to be stable at room temperature for up to 12 h with regard to DNA concentration and bacterial profiles,^171^ which may guide the clinician and/or owner as to optimal timing of collection relative to submission.

There is no ubiquitous protocol for sample collection, handling or processing, and significant variabilities exist between diagnostic tests, laboratories and veterinary practices. In order to maximise the utility of results, the authors therefore encourage the reader to liaise with their specific external laboratory where possible to determine the recommended or most appropriate sample collection, storage and courier requirements prior to submission.

**Figure:**
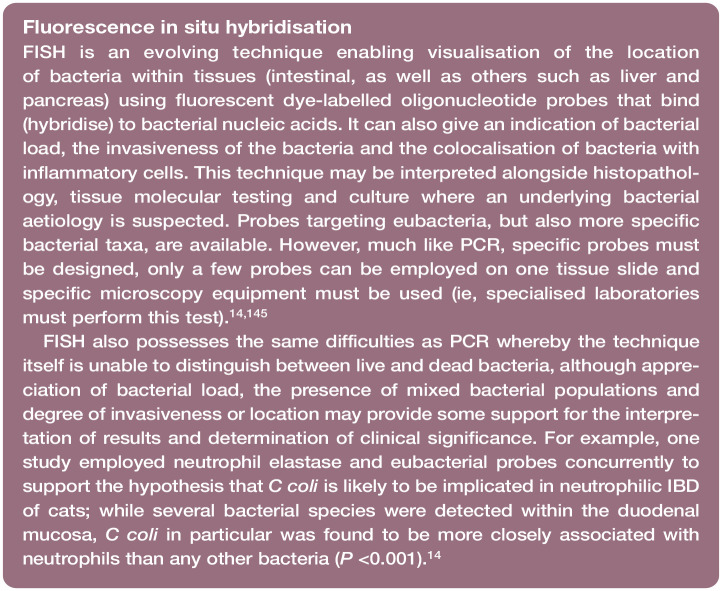


**Figure:**
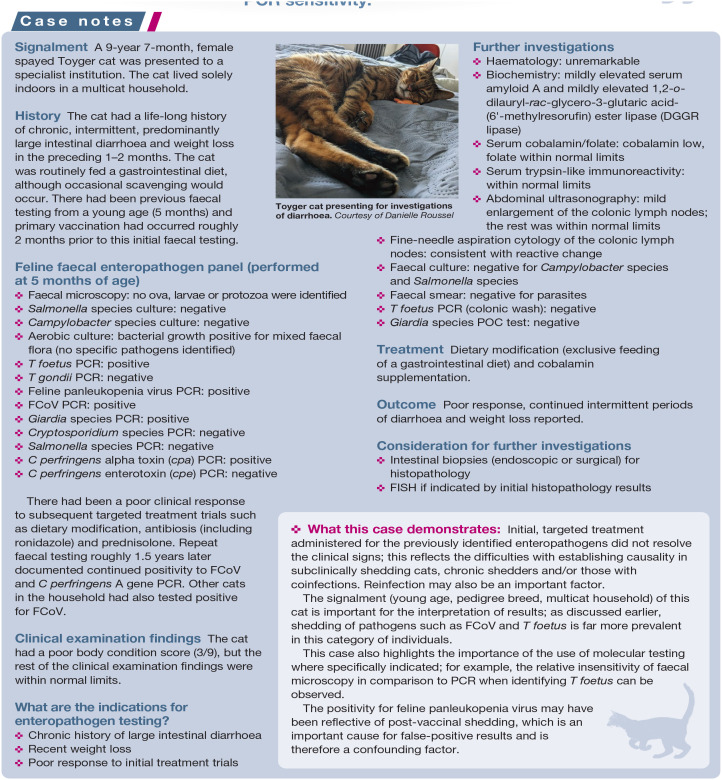


The signalment (young age, pedigree breed, multicat household) of this cat is important for the interpretation of results; as discussed earlier, shedding of pathogens such as FCoV and T foetus is far more prevalent in this category of individuals.

This case also highlights the importance of the use of molecular testing where specifically indicated; for example, the relative insensitivity of faecal microscopy in comparison to PCR when identifying T foetus can be observed.

The positivity for feline panleukopenia virus may have been reflective of post-vaccinal shedding, which is an important cause for false-positive results and is therefore a confounding factor.

## Key Points

✜ The prevalence rates reported for feline enteropathogens are affected by multiple factors, including signalment, individual patient factors (immunosuppression, prior antibiotic treatment), seasonality and, importantly, method of detection used and timing of testing.

✜ An understanding of the aetiopathogenesis of disease can greatly improve case management.

✜ Molecular diagnostics can be employed as detection methods in various enteropathogenic organisms, including Campylobacter species, Clostridium species, FCoV/FIP, T foetus and Giardia species, among others.

✜ Although PCR offers many benefits over conventional testing methods such as faecal culture or microscopy, it is incredibly important to be aware of the limitations and complicating factors of this technique when interpreting results.

✜ Molecular diagnostics for feline enteric pathogens are not as simple to interpret as it may initially seem, and these tests must be requested in patients only where it is indicated to do so.

✜ The decision to initiate targeted treatment should be assessed on a case-by-case basis and once the discussions outlined within this review have been taken into consideration.
